# Increased tumorigenesis associated with loss of the tumor suppressor gene *Cadm1*

**DOI:** 10.1186/1476-4598-11-29

**Published:** 2012-05-03

**Authors:** Louise van der Weyden, Mark J Arends, Alistair G Rust, George Poulogiannis, Rebecca E McIntyre, David J Adams

**Affiliations:** 1 Experimental Cancer Genetics, The Wellcome Trust Sanger Institute, Hinxton, Cambridge, CB10 1HH, UK; 2 Department of Pathology, University of Cambridge, Addenbrooke’s Hospital, Hills Road, Cambridge, CB2 2QQ, UK; 3 Division of Signal Transduction, Beth Israel Deaconess Medical Center, Department of Systems Biology, Harvard Medical School, Boston, MA, 02115, USA

**Keywords:** Cell adhesion molecule, Tumor suppressor, Transposon, Glucocorticoid, Cell junction

## Abstract

**Background:**

*CADM1* encodes an immunoglobulin superfamily (IGSF) cell adhesion molecule. Inactivation of *CADM1*, either by promoter hypermethylation or loss of heterozygosity, has been reported in a wide variety of tumor types, thus it has been postulated as a tumor suppressor gene.

**Findings:**

We show for the first time that *Cadm1* homozygous null mice die significantly faster than wildtype controls due to the spontaneous development of tumors at an earlier age and an increased tumor incidence of predominantly lymphomas, but also some solid tumors. Tumorigenesis was accelerated after irradiation of *Cadm1* mice, with the reduced latency in tumor formation suggesting there are genes that collaborate with loss of *Cadm1* in tumorigenesis. To identify these co-operating genetic events, we performed a *Sleeping Beauty* transposon-mediated insertional mutagenesis screen in *Cadm1* mice, and identified several common insertion sites (CIS) found specifically on a *Cadm1*-null background (and not wildtype background).

**Conclusion:**

We confirm that *Cadm1* is indeed a bona fide tumor suppressor gene and provide new insights into genetic partners that co-operate in tumorigenesis when *Cadm1*-expression is lost.

## Findings

Cell adhesion molecule 1 (*CADM1*; also known as *TSLC1, IGSF4, Necl-2, RA175SgIGSF, SynCAM1*) is member of the immunoglobulin superfamily of cell adhesion molecules (IGSF-CAMs) and is composed of an extracellular domain containing three immunoglobulin-like C2-type domains, a transmembrane domain and a short cytoplasmic tail [1]. The extracellular domain of CADM1 mediates the formation of homodimers or heterodimers with other CAM members, including Necl-1, CRTAM and Nectin-3 to regulate cell adhesion. The cytoplasmic domain of CADM1 interacts with the tumour-suppressor gene DAL-1 and the group of membrane-associated guanylate kinase (MAGuK) homologues, as well as being able to modulate the activation of small Rho GTPases, thus acting as a vital bridge between extracellular adhesion and intracellular signaling cascades. In addition, CADM1 can also modulate cell cycle progression and apoptosis [2,3].

Less than a decade since the discovery of *CADM1*, loss of its expression by promoter hypermethylation or loss of heterozygosity (LOH) has been reported in a wide variety of tumor types ( Additional file 2: Figure S1) and frequently correlates with advanced tumor stage (poor prognosis) and metastasis [3]. Studies in nude mice have demonstrated that re-expression of CADM1 suppresses *in vivo* tumorigenicity of non-small cell lung cancer and nasopharyngeal carcinoma cell lines [1,4,5]. In contrast, studies using *Cadm1* null (*Cadm1*^*−/−*^) mice have demonstrated important roles for Cadm1 in spermatogenesis/male fertility [6], behavior and motor performance [7], lens fiber cell architecture [8], and epidermal adhesion and wound repair [9]. However, there are no reports on the incidence of cancer in these mice and thus whether *Cadm1* is indeed a bona fide tumor suppressor gene.

**Figure 1:**
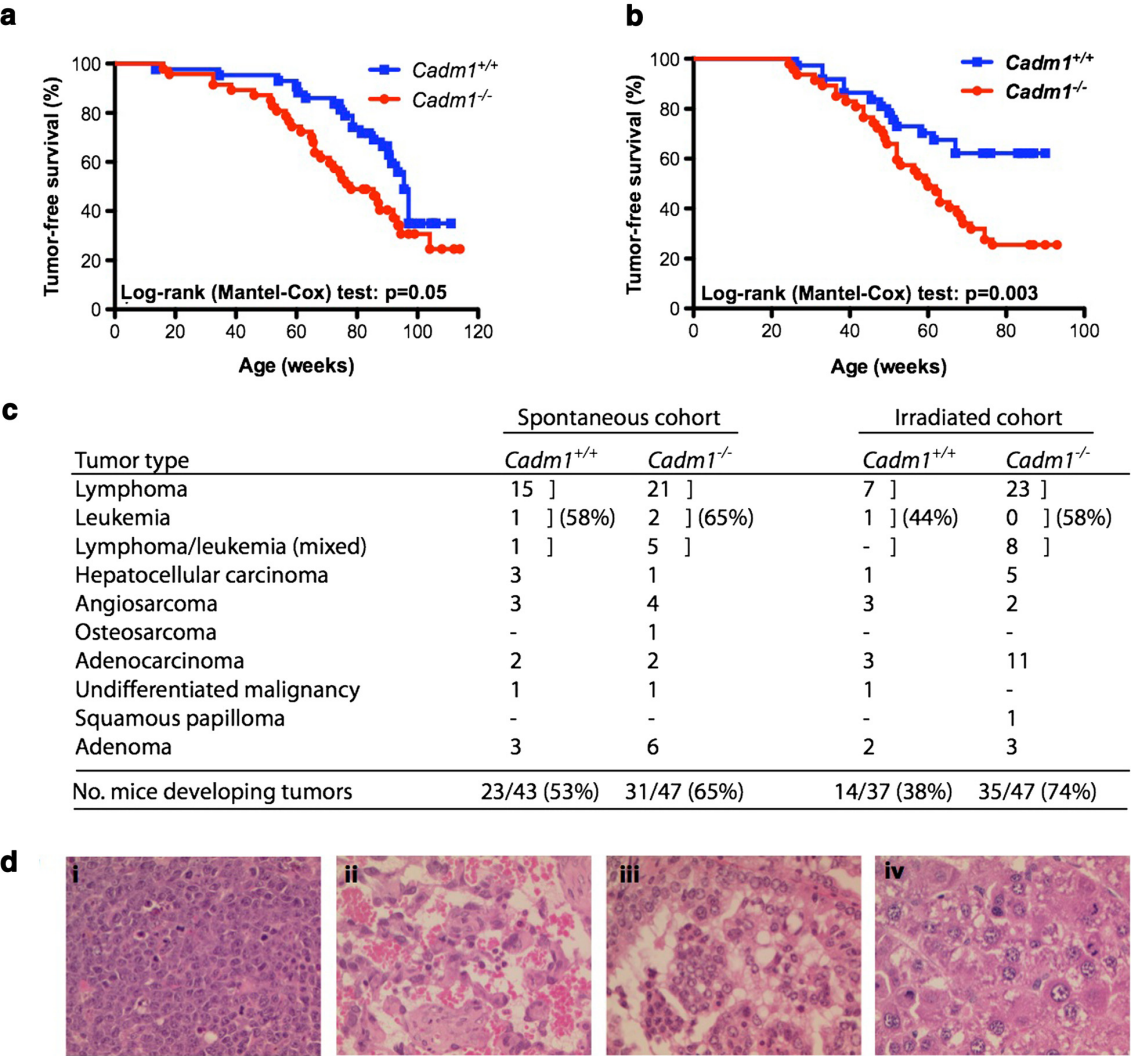
***Cadm1 *null mice succumb to tumor formation faster than their wildtype littermates. a. ***Cadm1* null mice (*Cadm1*^*−/−*^) and their wildtype littermates (*Cadm1*^*+/+*^) were put on tumor watch from birth, and monitored for the development of spontaneous tumor development. **b.***Cadm1*^*−/−*^ and *Cadm1*^*+/+*^ mice were whole body irradiated (3.5 Gy) at 6–8 weeks of age then placed on tumor watch, and monitored for the development of spontaneous tumor development. **c.** The decreased survival of *Cadm1* null mice was due to tumor formation, of a variety of types. **d.** Representative histological images of hematoxylin-eosin stained tumors from *Cadm1* null mice in the irradiated cohort, including (i) a splenic lymphoma, (ii) an angiosarcoma in the leg, (iii) a lung adenocarcinoma, and (iv) a hepatocellular carcinoma. All magnifications are x400.

We show here that *Cadm1*^−/−^ mice died significantly faster than their wildtype littermates (*Cadm1*^+/+^; average survival of 78 and 95 weeks of age for *Cadm1*^−/−^ and *Cadm1*^+/+^ mice, respectively) due to the spontaneous development of tumors at an earlier age (Log-rank (Mantel-Cox) test: p = 0.05; Figure 1a). When subjected to irradiation, *Cadm1*^−/−^ mice developed significantly more tumors than their wildtype littermates, and at an earlier age (62 and 81 weeks for *Cadm1*^−/−^ and *Cadm1*^+/+^ mice, respectively; Log-rank (Mantel-Cox) test: p = 0.003; Figure 1b). The predominant tumor type in both cohorts was lymphoma and/or leukemia (typically widely disseminated), although a number of solid tumors were also observed, including angiosarcoma, adenocarcinoma (of the lung, jaw or stomach) and hepatocellular carcinoma (Figure 1c-d). This is consistent with the frequent silencing of CADM1 observed in human cancer types, both of epithelial [2,3] and hematopoietic origin [10,11].

**Figure 2:**
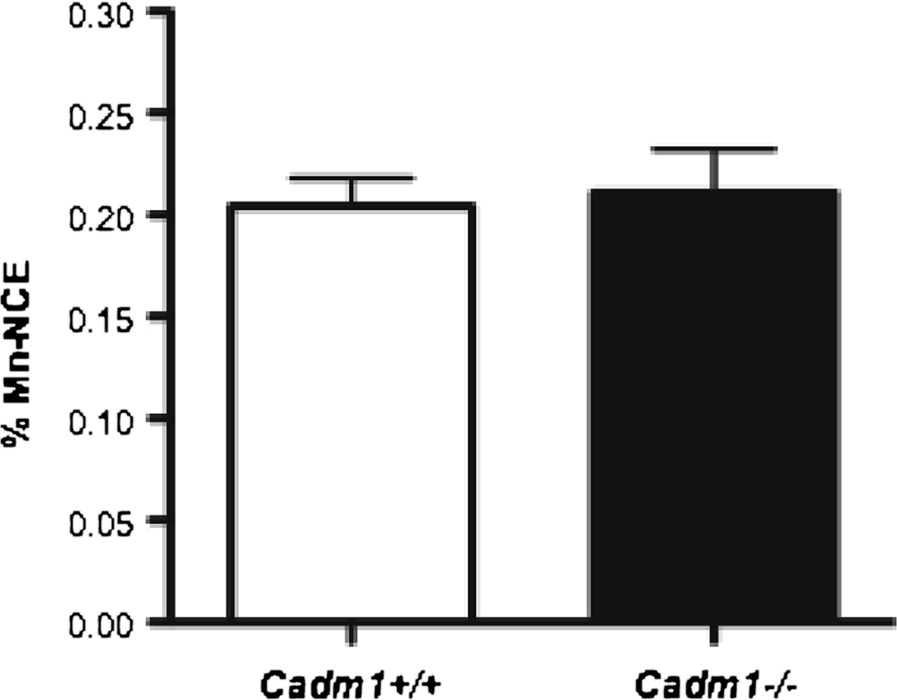
**Micronucleus assays on *Cadm1 *mice.** Peripheral blood was collected from *Cadm1 * null and wildtype mice at 6 to 7 weeks of age and stained with anti-CD71-FITC antibody and propidium iodide before being analyzed by flow cytometry. A minimum of 50,000 events were analyzed for each sample (n = 4–5 per genotype) and the data are represented as percentage of normochromatic erythrocytes possessing micronuclei.

**Figure 3:**
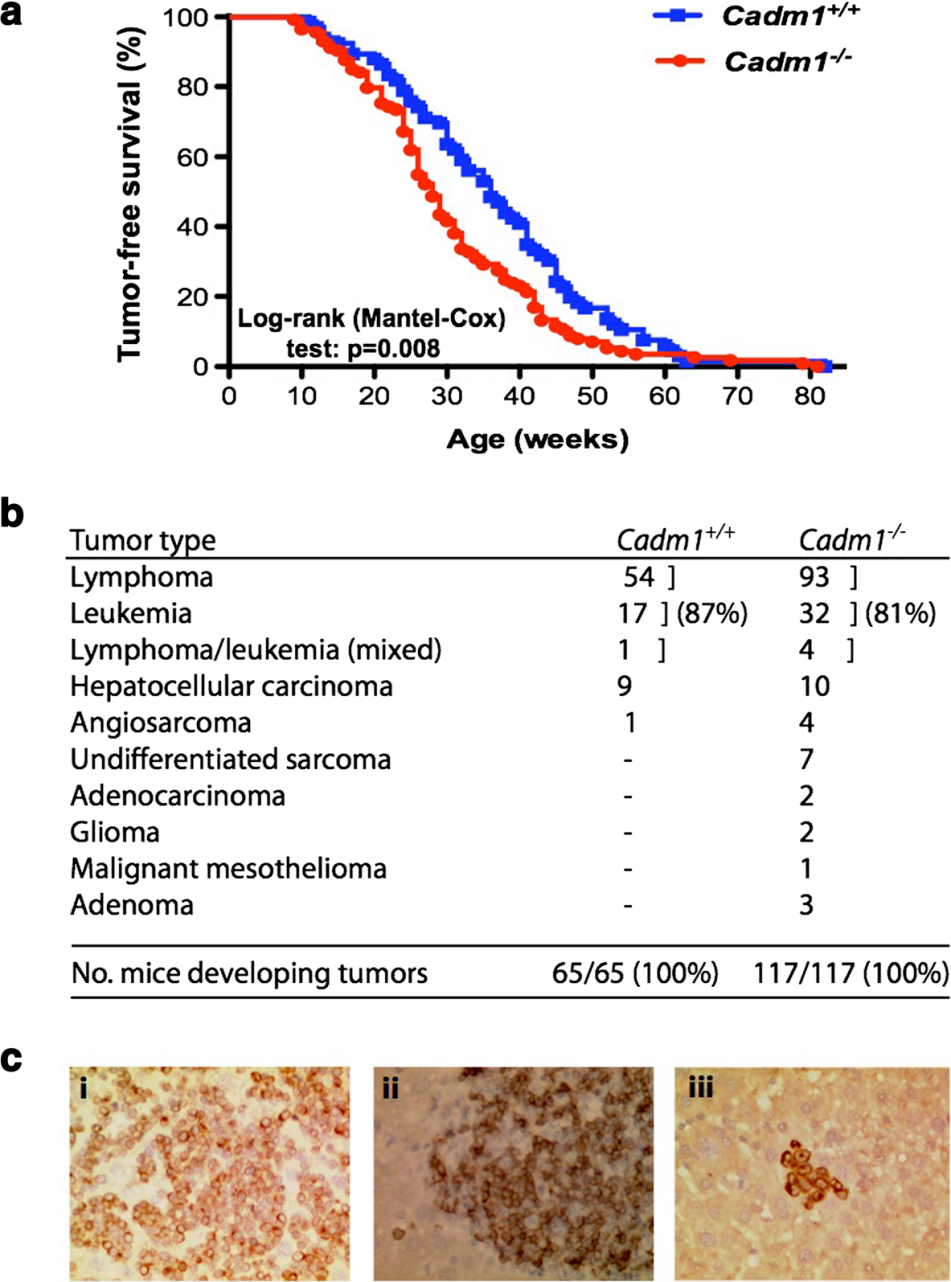
**Insertional mutagenesis using *Sleeping Beauty *transposons in *Cadm1 *mice. ***Cadm1 * mice were bred onto a genetic background that allowed for Sleeping Beauty (*SB*) transposon-mediated insertional mutagenesis to occur in the soma, and placed on tumor watch.** a. ***Cadm1 SB* null mice died significantly faster than their wildtype *SB * littermates. **b. ** This decreased survival of *Cadm1 SB* null mice was due to tumor formation, of a variety of types. **c.** Representative immunohistochemical images of (i) a lymphoma staining positive for CD3, (ii) a lymphoma staining positive for CD45R, (iii) and a leukemia staining positive for MPO. All magnifications are x400.

To assess whether loss of *Cadm1* resulted in increased genomic instability, we used the highly sensitive flow-cytometric micronucleus assay, which provides a quantitative measure of *in vivo* chromosome damage [12]. Micronuclei can arise from acentric chromosome fragments or whole chromosomes that have not been incorporated in the main nuclei at cell division. However, as shown in Figure 2*Cadm1* null mice did not show higher levels of micronuclei than wildtype littermates, suggesting that the absence of Cadm1 does not result in gross genomic instability. To gain mechanistic insights into how loss of *Cadm1* results in increased tumorigenesis, we performed an insertional mutagenesis screen using the *Sleeping Beauty* (*SB*) transposon in *Cadm1* mice to identify genes that co-operate with loss of *Cadm1* in tumor formation. *Cadm1*^*−/−*^ mice with *SB* transposition occurring (i.e., on a *T2/Onc*^*+/Tg*^*, Rosa26*^*+/SB11*^ background; *Cadm1*^*−/−*^*SB* mice) developed tumors significantly faster than their wildtype *SB* littermates (average lifespan of 28 and 36 weeks for *Cadm1*^−/−^ and *Cadm1*^+/+^ mice, respectively; Log-rank (Mantel-Cox) test: p = 0.008; Figure 3a). As previously reported for the *T2/Onc* transposon [13], *SB* mice typically developed lymphoma and/or leukemia (due to the use of the murine stem cell virus promoter which is preferentially expressed in cells of the hematopoietic compartment), although a small proportion of mice did develop additional tumors, typically hepatocellular carcinoma (Figure 3b). Immunohistochemical analysis of a selection of the *SB*-induced lymphomas and/or leukemias (Figure 3c) showed the predominant disease subtype was a CD3-positive T-cell lymphoma (51/108, 47%), followed by myeloperoxidase-positive high-grade leukemia (27/108, 25%), poorly differentiated lymphoma not staining positively for either T-cell (CD3) or B-cell (CD45R) antigens (19/108, 18%) and CD45R-positive B-cell lymphoma (11/108, 10%).

**Figure 4:**
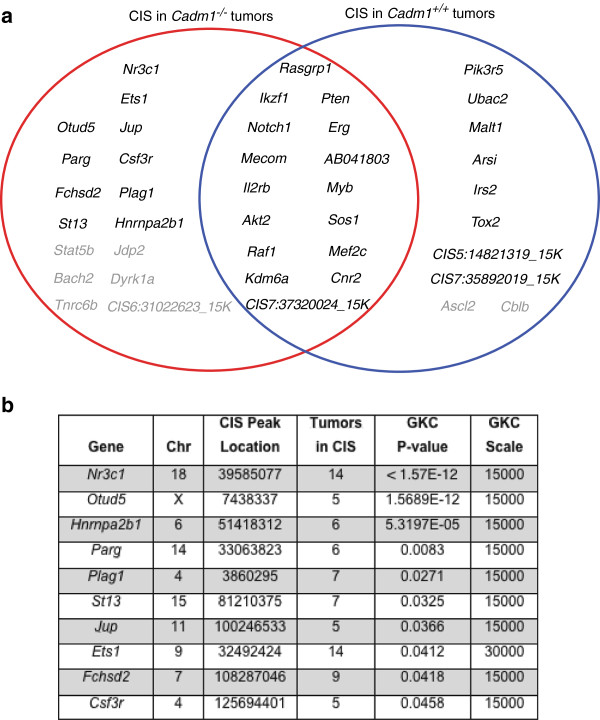
**Common insertion sites (CIS) found in the leukemia/lymphoma cases from *Cadm1 *mice.** Gaussian kernel convolution (GKC) CIS were called as detailed in the Materials and Methods (Additional file 1). **a.** Venn diagram showing the CIS found in the leukemia/lymphoma cases from *Cadm1 *^*+/+ *^ (blue circle) and *Cadm1 *^*−/−*^ (red circle) mice. CIS shown in black are those with a genome-wide adjusted P-value of <0.05, and CIS shown in grey are those also present in the opposite genotype but at a genome-wide adjusted P-value of >0.05. CIS that are not located within ± 150 K base pairs of a gene are given the label ‘CIS’ followed by the chromosome and the peak location of the Gaussian kernel. **b.** Details of the CIS (with a genome-wide adjusted P-value of <0.05) found only in tumors from *Cadm1 *^*−/− *^ mice, thus representing *Cadm1* null-specific CIS. ‘Tumors in CIS’ is the number of individual tumor samples (mice) that contained insertions in the gene/CIS region.

Given lymphoma and/or leukemia (hereafter collectively referred to as ‘lymphoma’) was the most common tumor type, only these tumors from the *SB* cohort were used for analysis of somatically mutated genes (to ensure sufficient insertion sites to allow statistical power to identify ‘common insertion sites’ (CIS); genomic regions with a higher density of insertion sites than expected by chance). Genomic DNA extracted from lymphomatous tissues of the *SB* mice (spleen, thymus, liver or lymph node) was used in a splinkerette PCR reaction to produce barcoded PCR products that were subsequently pooled and directly sequenced on the 454 GS-FLX platform [14]. This generated 876,117 sequence reads, of which 46.93% unambiguously aligned to the mouse genome. Using a previously developed computational pipeline to trim, map, and annotate each sequence read [14], we were able to identify 47,220 unique (non-redundant) integrations or insertion sites. We used the Gaussian kernel convolution (GKC) algorithm to determine statistically significant CIS, which were then assigned to genes as described previously [14]. Unique GKC CIS regions/genes were identified from 73 *Cadm1*^*+/+*^ and 117 *Cadm1*^*−/−*^ lymphomatous mice as two independent groups (Figure 4a). The two groups of CIS calls (using a genome-wide P value of cut off of <0.1) were compared to generate a list of CIS found only in the *Cadm1*^*−/−*^ mice. Then, increasing the stringency to include only those CIS with a genome-wide adjusted P-value of <0.05, gave us a final list of 10 ‘*Cadm1*-null specific’ CIS (Figure 4b).

The most statistically significant CIS was in the *Nr3c1* gene, which encodes the glucocorticoid receptor (GR), and the insertions would be predicted to have a loss-of-function effect on *Nr3c1*, consistent with the finding of promoter hypermethylation or mono-allelic deletion of *NR3C1* in several cancer types including colo-rectal cancer (CRC) [15] and leukemia [16], respectively. Glucocorticoids (GCs), which bind the GR and allow it to translocate to the nucleus and modulate gene expression, are effective inhibitors of proliferation and tumorigenesis and routinely used in treating T-cell acute lymphoblastic leukemia (T-ALL) [16]. Interestingly, three of the other nine CIS genes identified are known interactors/regulators of the GR. *St13* encodes the Hsp70-interacting protein that is involved in the assembly process of the GR, and *ST13* mRNA and protein levels are down-regulated in CRC [17]. The *Ets1* proto-oncogene is a transcription factor that can act as a “molecular switch” for auto-regulation of the GR promoter, and high *ETS1* expression predicts poor prognosis in patients with ovarian cancer [18]. The *Csf3r* encodes the cell-surface granulocyte colony-stimulating factor (G-CSF) receptor, and activated GR can synergize with G-CSF signals [19].

Cell junctions including tight junctions, adherens junctions and desmosomes, consist of multi-protein complexes that provide contact between neighboring cells or between a cell and the extracellular matrix and as such play important roles in regulation of cell proliferation and differentiation, as well as cancer [20]. Like CADM1, two of our CIS genes are part of these multi-protein complexes. *Fchsd2* encodes the FCH and double SH3 domains protein 2 (FCHSD2), which binds to epithelial junction MAGuKs, specifically MAGI-1 and CASK [21]; CADM1 has been shown to interact with several MAGuK members, including CASK [22]. *Jup* encodes junction plakoglobin (JUP), which complexes with numerous other desmosomal proteins (including cadherins, desmogleins and desmocollins) [23]), and was recently shown to be expressed on the surface of colorectal cancer cells associated with high metastatic potential [24].

Finally, it is interesting to note that in addition to ‘*Cadm1* null-specific’ CIS, we also identified CIS that were only found in tumors from wildtype mice (i.e., not found in *Cadm1* null tumors). These CIS represent loci that are mutated in the process of tumorigenesis in the presence of an intact Cadm1 signaling pathway, and whose contribution to tumorigenesis is potentially rendered obsolete in the absence of *Cadm1*. Some of these genes, such as *Pik3r5* and *Malt1*, have also been identified as CIS in leukemia/lymphomas from wildtype mice in other *Sleeping Beauty* transposon screens we have performed (unpublished data). Several CIS genes including *PtenNotch1* and *Erg* are mutated in both wildtype and *Cadm1* null tumors suggesting that mutation of these genes can contribute to tumorigenesis regardless of *Cadm1* status [25].

Thus we have shown that *CADM1* is a bona fide tumor suppressor gene, and loss of *Cadm1* results in an increased tumor incidence. Our insertional mutagenesis screen provides new insights into *Cadm1*-mediated tumor suppression by identifying genes that co-operate with loss of *Cadm1* in lymphomagenesis, in particular those regulating glucocorticoid signaling and cell junctions.

## Competing interests

The authors declare they have no competing interests.

## Author’s contributions

LvdW and DJA designed the experiments and performed the animal work; MJA performed the histopathological and immunohistopathological analysis; AGR and GP performed the bioinformatic statistical analysis; REM performed the micronucleus assay; LvdW wrote the manuscript with comments from all authors; all authors read and approved the final version of the manuscript.

## Supplementary Material

Additional file 1 **Supplementary information**. Materials and Methods. Reference list [26]. Click here for file

Additional file 2 **Figure S1.Analysis of *CADM1 *expression across different tumor types. A.** Box plots showing tumor types with significantly lower *CADM1* expression in cancer versus normal tissues in at least three independent microarray datasets. **B, C. ** Ranked *CADM1* expression in a dataset of lung adenocarcinomas and Kaplan-Meier survival curves comparing disease-free survival between cases with the lowest (<25th percentile) vs. highest (>25th percentile) *CADM1* expression (P = 2.7x10^-8^, log-rank test). **D.** Details of the microarray datasets used [27-31]. Click here for file
