# Supplementary material for: Increased tumorigenesis associated with loss of the tumor suppressor gene Cadm1
Source: Mol Cancer. 2012 May 3;11:29. doi: 10.1186/1476-4598-11-29 (PMC3489691; doi:10.1186/1476-4598-11-29)
Supplement: Additional file 1 — Supplementary information. Materials and Methods. Reference list [26]. [file 1476-4598-11-29-S1.docx]

**Supplementary Information:**

**Materials and methods**

***Animals:*** Generation and genotyping of *Cadm1* null mice (Tslc1^Brdm2^) [6], mice carrying the *SB* transposon array (*T2/Onc*) [13], and mice carrying the *SB* transposase (*Rosa26^SB11^*) [25] have been described previously. Whole body irradiation was performed at 3.5 Gy on 6-8 week old mice. All mice were on a mixed 129/Sv-C57BL/6J background and housed in accordance with Home Office regulations (UK).

***Histology and immunohistochemistry:*** Tissues were fixed in 10% neutral-buffered formalin (NBF) at room temperature overnight. Samples were then transferred to 50% ethanol, embedded in paraffin, sectioned and stained with hematoxylin and eosin. Immunohistochemistry was performed on formalin-fixed, paraffin-embedded tissue sections that had undergone antigen retrieval (microwaving in citrate buffer pH 6 for 20 min) using antibodies for CD3 (clone SP7; Abcam, Cambridge, UK), CD45R/B220 (clone RA3-6B2, R&D systems) and MPO (DAKO, Ely, UK). Immunohistochemical signal was detected by secondary biotinylated goat anti-rabbit antibody (Vector Laboratories, Burlingame, CA), followed by Vectorstain Elite ABC kit (Vector Laboratories) according to the manufacturer’s instructions.

***Micronucleus assay:*** Blood was collected from wildtype and *Cadm1* null 6- to 7-week-old mice by cardiac puncture during terminal anesthesia and immediately fixed in methanol overnight at −80°C. The cells were stained with rat anti-mouse CD71-FITC (Southern Biotech, Birmingham, AL, USA) and propidium iodide (50 μg/ml; Sigma, Dorset, England). Flow cytometry was performed on the samples with fifty thousand red blood cells examined from each animal (n=4-5 per genotype). Analysis of the data performed using FlowJo 5.4+ software (TreeStar, Ashland, OR, USA).

***Isolation & statistical analysis of transposon insertion sites****:* Isolation of the transposon insertion sites from the tumours was performed using splinkerette PCR to produce barcoded PCR products that were pooled and sequenced on 454 GS-FLX sequencers (Roche platform) over four separate lanes as described previously [14]. Processing of 454 reads, identification of insertion sites, and Gaussian Kernel Convolution statistical methods used to identify common insertion sites (CIS) have been described previously [14]. Any CIS on mouse chromosome 1 were not reported (as the ‘donor chromosome’ is automatically excluded from analysis due to the phenomenon of ‘local hopping’) [14]. Genotype-specific CIS analysis was individually performed on the two different tumor genotypes (*Cadm1* null and *Cadm1* wildtype tumors) using a genome-wide adjusted P-value cut-off of P<0.1 to ensure stringency when detecting shared CIS (such that CIS found in each genotype were compared to CIS that were marginally on the wrong side of the 0.05 cut-off  (up to a significance of 0.1) and would have otherwise been missed). ‘*Cadm1* null-specific CIS’ were defined as those with a genome-wide P value of <0.05 that were not identified as CIS in the *Cadm1* wildtype tumors.

***Bioinformatic meta-analysis of CADM1 expression*:** Microarray expression data from four independent data sets were downloaded from the Oncomine repository (<http://www.oncomine.org/>) to examine the relative mRNA expression levels of *CADM1* between normal and cancer samples. The distributions of log2 median-centered signal intensities were plotted using boxplots and differential gene expression was computed using the Welch two sample t-test, which is appropriate for subsets of unequal variances. Only tumor sets showing the same differential mode of expression in at least three independent datasets were included in this analysis. To correlate gene expression of *CADM1* with patient survival, a univariate cox proportional hazard regression model was applied to a lung adenocarcinoma dataset of n=443 samples and the Likelihood ratio test, Wald test, and Score (logrank) test were all used to compute the P value. To visualize the result obtained from the survival analysis, the samples were ranked according to *CADM1* gene expression and Kaplan-Meier survival curves were plotted for adenocarcinomas with the lowest (<25th percentile) versus highest (>25th percentile) RUNX2 expression giving a P value of 2.7x10^-8^ (logrank test).
