# Supplementary figures and images for: Increased tumorigenesis associated with loss of the tumor suppressor gene Cadm1
Source: Mol Cancer. 2012 May 3;11:29. doi: 10.1186/1476-4598-11-29 (PMC3489691; doi:10.1186/1476-4598-11-29)

Figure S1

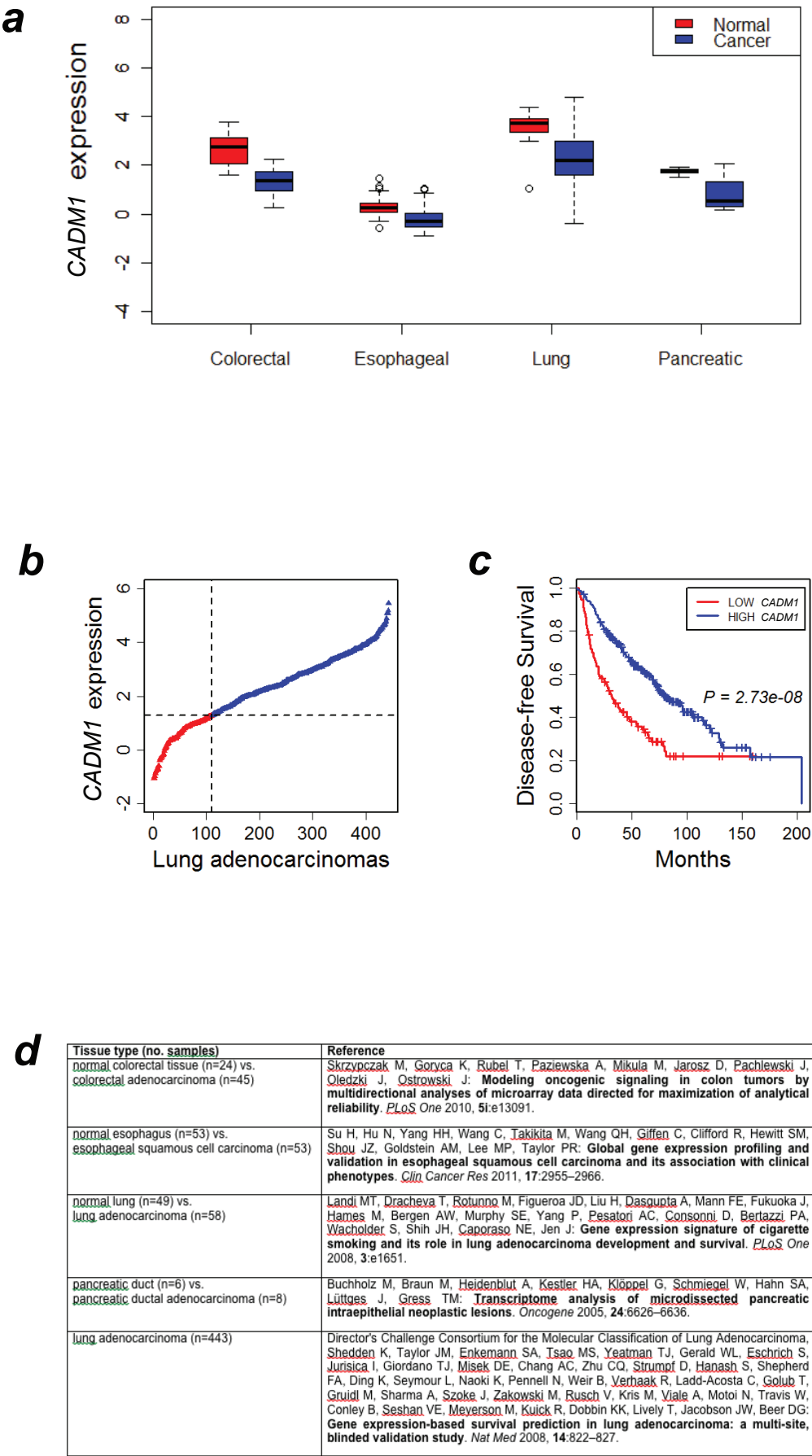

Supplement: Additional file 2 — Figure S1.Analysis of CADM1 expression across different tumor types. A. Box plots showing tumor types with significantly lower CADM1 expression in cancer versus normal tissues in at least three independent microarray datasets. B, C. Ranked CADM1 expression in a dataset of lung adenocarcinomas and Kaplan-Meier survival curves comparing disease-free survival between cases with the lowest (<25th percentile) vs. highest (>25th percentile) CADM1 expression (P = 2.7x10^-8^, log-rank test). D. Details of the microarray datasets used [27-31]. [file 1476-4598-11-29-S2.pdf]
